# Assessment of practices and awareness regarding the disposal of unwanted pharmaceutical products among community pharmacies: a cross-sectional study in Palestine

**DOI:** 10.1186/s12913-023-09888-5

**Published:** 2023-09-27

**Authors:** Lamees L. Nairat, Noor A. Abahri, Yosr A. Hamdan, Razan T. Abdel-khaliq, Sawsan M. Odeh, Shatha Abutaha, Samah W. Al-Jabi, Amer A. Koni, Amani S. Abushanab, Sa’ed H. Zyoud

**Affiliations:** 1 Department of Clinical and Community Pharmacy, College of Medicine and Health Sciences, An-Najah National University, Nablus, 44839 Palestine; 2 Department of Medicine, Faculty of Medicine and Health Sciences, An-Najah National University, Nablus, 44839 Palestine; 3 Division of Clinical Pharmacy, Department of Hematology and Oncology, An-Najah National University Hospital, Nablus, 44839 Palestine; 4 Poison Control and Drug Information Center (PCDIC), College of Medicine and Health Sciences, An-Najah National University, Nablus, 44839 Palestine; 5 Clinical Research Center, An-Najah National University Hospital, Nablus, 44839 Palestine

**Keywords:** Awareness, Practices, Community pharmacists, Disposal, Unwanted pharmaceutical products

## Abstract

**Background:**

The improper disposal of pharmaceutical preparations substantially threatens human health and environmental safety. Pharmacists are responsible for properly disposing of unwanted medications and educating patients about how to do so themselves. This study aimed to assess community pharmacists’ knowledge, determine their views on how to dispose of unwanted pharmaceuticals, and assess the extent to which they realize that it is their responsibility to guide patients toward the safe disposal of expired medications.

**Methods:**

A descriptive cross-sectional study was conducted between December 2021 and April 2022 among 400 practicing pharmacists who were chosen to participate by random cluster sampling. Community pharmacists’ practices, awareness, and beliefs about disposing of unused drugs were evaluated. The Statistical Package for Social Sciences (IBM-SPSS) version 21 was used for data entry and analysis.

**Results:**

Of 400 pharmacists, 348 stated that they did not participate in courses on the safe disposal of unwanted medications. Disposal of drugs in the garbage, an unsafe method, was very frequently recommended by pharmacists to patients, especially regarding inhalers, antibiotics, hormonal drugs, and solid and semisolid drugs. However, many pharmacists advised patients to return their hormonal, category B, and category C drugs to the pharmacy. A total of 61.3% of pharmacists agreed and 26% strongly agreed that unsafe disposal of drugs negatively affects the environment. A total of 54.3% of the participants agreed that improper disposal of antibiotics might be a reason for increased antimicrobial resistance, and 54.5% of them agreed that improper disposal of hormonal drugs might contribute to the development of certain cancers. A total of 80.3% of the participants perceived that most unwanted drugs in pharmacies were those returned from patients. A total of 97.3% of the participants supported establishing a drug disposal system, with 77.5% choosing to have the district health board responsible for funding this system. A total of 48.5% of the participants indicated that a lack of education and awareness on the issue of getting rid of unused drugs constitutes a challenge to the safe disposal of medicines, and 66% of them said that a lack of law enforcement constitutes another challenge. A total of 95.5% of the participants agreed or strongly agreed that good training for health sector workers and organizing workshops to develop knowledge on this subject would improve practices. A total of 93.3% supported distributing educational brochures, and 92.8% supported placing special containers in every pharmacy to collect unwanted drugs.

**Conclusions:**

Most pharmacists in our study returned drugs to manufacturing companies and stores, and few followed the correct methods of incineration and return of drugs to the Ministry of Health. Current data emphasize the issue of improper disposal of medicine in Palestine and the need for improved education among healthcare workers.

## Background

The appropriate disposal of unused drugs is crucial for the safety of humans and the environment and to prevent potentially hazardous consequences. In addition, different drugs in the environment through improper disposal can adversely affect humans and animals. For example, the United States Food and Drug Administration reported fatal cases among children due to unintended exposure to fentanyl transdermal patches [1–3].

Pharmacists play an important role in safe medication disposal, as they are considered one of the ways to provide patients with the best care. Many countries have established programs to collect drugs and safely dispose of unused medications [4, 5]. The United States FDA developed guidelines providing advice on appropriate disposal practices that also support the urgent need for instructions of safe disposal instructions on the medication label. In addition, the United States FDA developed a program to collect and appropriately dispose of unwanted pharmaceutical preparations [3].

In the United Kingdom, policies have been formulated asking community pharmacies to collect unwanted drugs from the public where other health agencies will be responsible for the appropriate disposal process [6]. The Australian government conducted a similar approach, necessitating community pharmacies to collect unwanted medications [7]. In contrast, in Palestine, there are no policy guidelines for incineration for the disposal of unwanted pharmaceutical preparations. To establish such a program for collecting drugs back by community pharmacists, data about practices and awareness regarding the disposal of unused pharmaceutical products among pharmacists are needed. This cross-sectional study sheds light on awareness and practices of unwanted pharmaceutical products among community pharmacists in Palestine.

Unwanted medications are those that have gone unused or have expired, spilled, and contaminated pharmaceuticals and drugs, vaccines, and sera that are no longer required and need to be disposed of appropriately [8]. Sources of these unwanted medications include both households and healthcare facilities. Regarding households, unwanted medications can accumulate due to excessive prescription by physicians, poor adherence to medications, or over-the-counter medications that they buy and then expire [9]. Hospitals and healthcare facilities are also prominent sources of unwanted medications, with waste generated from partially used or unused drugs, outdated drugs, and personal patient medications [10]. Absent medications must be disposed of properly from households or health facilities. However, when unwanted medications accumulate, they are typically improperly disposed of in the trash or the sewage system, thus entering the environment and posing a risk to the well-being of wildlife and people [5].

Several reports have provided evidence of the presence of drugs and their metabolites in the environment, contaminating soil and water [11, 12]. It has been shown that approximately 10% of pharmaceutical products have a significant potential environmental risk, especially hormonal drugs, analgesics, antidepressants, antineoplastics, and antibiotics [13]. Chronic exposure to these chemicals threatens the health of humans and animals. It has been shown that ethinylestradiol contributes to feminization of male fish in certain rivers [14]. Antidepressants in surface water have been shown to alter animal behaviors that are known to have evolutionary and ecological consequences [15]. Furthermore, the disposal of antibiotics in the sewage system may contribute to the development of antibiotic resistance among bacteria by selective pressure [16]. This highlights the need to minimize the quantity of pharmaceuticals in the environment with all available strategies.

In many countries, there is much confusion about the proper methods to dispose of unwanted medications, especially where local guidelines are lacking or nonexistent [17]. In general, the most common method for household disposal is disposal in the garbage, while flushing some drugs down the toilet still takes place in countries such as the United States and New Zealand [18]. A previous study found that most people throw medications in the garbage, and less than 14% return unused drugs [19]. Therefore, the US Food and Drug Administration (FDA) recommends drug take-back programs as the best method to dispose of unwanted medications [20]. When this is not feasible, and the medication is on the FDA’s ‘flush list’, they recommend flushing these drugs immediately down the sink or toilet [21]. Otherwise, they provide instructions on safely discarding drugs in the household trash.

Drug take-back programs provide a safe, legal, and environmentally friendly avenue to dispose of medications from homes [20, 22]. The public is encouraged to return unwanted medications to community pharmacies, where approved agents collect and properly dispose of these drugs. Take-back programs are implemented in approximately 30 countries, and pharmacies play a central role in these programs [5]. When properly implemented and on a wide scale, these programs have the potential to significantly reduce the negative environmental impact caused by improper medication disposal [23]. This study focuses on Palestine, a developing nation without a drug take-back program and with no clear guidelines regarding the disposal of unwanted medications. A previous pilot study in Nablus-Palestine found that 67% of patients and healthcare professionals dispose of unwanted pharmaceuticals in normal household bins, and the study emphasizes the need for guidelines in pharmacies and hospitals for proper disposal of these unwanted products [24]. Another study conducted at hospitals and homes in the Gaza Strip showed inadequate handling of unwanted medicines [25].

Educational interventions, even brief interventions, are effective in changing pharmacists’ attitudes and knowledge of drug disposal practices [26]. This emphasizes the need to educate healthcare workers and pharmacists on proper medication disposal. Pharmacists interact a great deal with patients and influence not only how they use their medications but also how they dispose of them. Therefore, accessible healthcare providers are in a prime position not only to dispose of unwanted drugs in their pharmacies but also to advise patients about the proper disposal of medications within the household. This study aimed to evaluate the methods community pharmacists use to dispose of unwanted drugs, the methods they recommend to patients, and their awareness of the environmental hazards of improper drug disposal. We also aimed to evaluate their views on recommendations to improve safe drug disposal practices, including funding for drug-disposal programs and perceived challenges facing drug disposal processes.

## Methods

### Study design

A descriptive cross-sectional survey of community pharmacists was conducted in Palestine between December 2021 and April 2022.

### Study area and study population

The study included licensed professionals and registered pharmacists who worked in community pharmacies throughout the West Bank.

### Sample size and sampling technique

According to the last report by the Palestinian Ministry of Health, the number of registered pharmacists in Palestine is 5655. The sample size was calculated using an online Raosoft sample size calculator. The minimum effective sample size was 360, assuming a 5% error margin, a 95% confidence interval (CI), and a response distribution of 50%. ‘A sample of 400 pharmacists from community pharmacies from all West Bank areas were selected and chosen conveniently. The data were collected by five pharmacy students who were in their final year of study through face-to-face interviews.

### Inclusion and exclusion criteria

This study included practicing pharmacists of all academic degrees who worked in community pharmacies in the West Bank, aged 23 years and older. Pharmacists’ assistants were excluded from the study.

### Data collection instrument

The questionnaire was adopted from several previous studies and consisted of 36 questions divided into six sections [27–32]. The first section was concerned with demographic data, and participants were asked if they had participated in educational courses or workshops on this topic. The second section evaluated the practice of pharmacists on how to dispose of unwanted medicines (including expired medications) in the pharmacy, providing them with several options to answer, with the possibility of choosing more than one answer. The third section evaluated whether pharmacists could provide sound advice and direct patients to the safest way to dispose of a variety of drugs, including solid, semisolid, liquid, expired class B controlled drugs (i.e., morphine, methylphenidate), expired class C controlled drugs (i.e., codeine, diazepam), hormonal drugs, inhalers, and antibiotics.

The fourth section evaluated pharmacists’ convictions about the environmental dangers of improper disposal of expired medicines, particularly antibiotics and hormonal medications. Their feelings of responsibility towards the environment and other living creatures were assessed, in addition to what they perceived as the sources of unwanted medications. This section was evaluated through six questions on the 5-Linkert scale, with a higher score indicating good awareness.

Section five gauged pharmacists’ opinions on whether Palestine needs a national drug disposal system and who they believe should be responsible for financing it. Finally, section six evaluated pharmacists’ views on recommendations to improve safe drug disposal practices and their opinions on common challenges facing disposal processes.

### Validity and reliability

A group of three specialist pharmacists (including a linguist) assessed the final questionnaire’s face and content validity. The questionnaire was offered in Arabic. They evaluated the organization, clinical terminology, completeness, appropriateness, logical sequence, and accuracy of the statements and modified some questions as needed. The questionnaire was piloted among 10 patients to test its readability and reliability. However, the results of the pilot testing were not included in the final data analysis. The survey instrument was improved based on the feedback received during the pilot review. The Cronbach’s alpha for the awareness questions was 0.688 and 0.643 for pharmacists’ views on recommendations to improve safe drug-disposal practices.

### Ethical issues

Our research was approved by the Institutional Review Board (IRB) of An-Najah National University (Ref: Med Sep. 2021/73). Furthermore, the pharmacists obtained informed consent verbally before participating in the survey. Finally, we described the study goals to the pharmacists and asked them to participate in the study.

### Statistical analysis

Data were entered and analysed using the statistical package for Social Sciences (IBM-SPSS) program, version 21. Categorical variables are expressed as frequencies and percentages, while continuous variables are described as medians and IQRs (interquartile ranges). In addition, correlations between pharmacists’ characteristics and awareness scores were analysed using Mann‒Whitney U and Kruskal‒Wallis tests. A P value of less than 0.05 was considered statistically significant.

## Results

### Demographic data of pharmacists who participated in the study

Four hundred pharmacists completed the questionnaire, 287 women (71.8%) and 212 (53%) were married. The median age (IQR) of the participants was 27 (24–30) years, and the majority lived in cities (249, 62.3%). The majority of the participants were from Jenin (166, 41.5%), followed by Nablus (110, 27.5%) (Table 1).

**Table 1:** Characteristics of pharmacists

Variable	n (%)
**Sex**	
Male	113 (28.3)
Female	287 (71.8)
**Age (years), median and interquartile range**	27 (24–30)
**Pharmacy location**	
City	249 (62.3)
Village	143 (35.8)
Refugee camp	7 (1.8)
**Job title**	
Pharmacist	342 (85.5)
Responsible pharmacist	58 (14.5)
**Pharmacists’ residence**	
City	220 (53.7)
Village	174 (42.4)
Refugee camp	6 (1.5)
**City**	
Bethlehem	3 (0.8)
Nablus	110 (27.5)
Ramallah	13 (3.3)
Jerusalem	2 (0.5)
Jenin	166 (41.5)
Tulkarem	14 (3.5)
Salfit	7 (1.8)
Qalqilya	42 (10.5)
Tubas	7 (1.8)
Hebron	26 (6.5)
Other	10 (2.5)
**Marital status**	
Married	212 (53)
Single	188 (47)
**Academic qualifications**	
Bachelor of pharmacy	322 (80.5)
Doctor of pharmacy	63 (15.8)
Higher education (post-grad)	15 (3.8)
**Country of graduation**	
Palestine	358 (89.5)
Abroad	42 (10.5)
**Monthly income**	
Less than 400 US dollar	35 (8.8)
400–800 US dollar	300 (75)
Over 800 US dollar	65 (16.3)
**Years of practice, median and IQR**	3 (2–6)
**Previous participation in courses on proper drug disposal methods**	
Yes	52 (13)
No	348 (87)
**Total**	**400 (100)**

A total of 322 (80.5%) had only a bachelor of pharmacy degree, 63 (15.8%) were Doctors of Pharmacy, and 15 (3.8%) had pursued postgraduate higher education. A total of 358 (89.5%) of these pharmacists studied in Palestine, while 42 (10.5%) had studied abroad. Sixty-five (16.3%) participants earned more than 800 US dollars per month, and 53 (8.8%) earned less than 400 US dollars per month. Only 52 participants (13%) had previously participated in a course on proper drug disposal methods (Table 1).

### Methods of disposal of unused/unwanted drugs in the pharmacy

Most pharmacists in our study (293, 73.3%) disposed of unwanted drugs by returning them to manufacturing companies and warehouses. Approximately half of the pharmacists and one-fifth disposed of pharmaceutical products via environmentally unfriendly routes, such as the trash and sink, respectively. Other commonly used methods were drug disposal in a drug take-back box (141, 35.3%) and returning the drugs to the Ministry of Health (83, 20.8%) (Table 2).

**Table 2:** Methods of disposal of unused/unwanted drugs in the pharmacy

	n (%)
**Method of disposal**	
Via Trash	193 (48.3)
Via Toilet	14 (3.5)
Via Sink	90 (22.5)
Via Incineration	23 (5.8)
In a drug take-back box	141 (35.3)
Returning to companies/warehouses	293 (73.3)
Return to the Ministry of Health	83 (20.8)
Other	7 (1.8)
**Solid drugs**	
Trash	252 (63)
Toilet	14 (3.5)
Sink	1 (0.3)
Incineration	11 (2.8)
Returning to pharmacy	78 (19.5)
Donation to hospitals	16 (4)
Giving to friends/family	6 (1.5)
None recommended (I don’t know)	19 (4.8)
Other	3 (0.8)
**Total**	**400 (100)**
**Semisolid drugs**	
Trash	196 (49)
Toilet	8 (2)
Sink	85 (21.3)
Incineration	4 (1)
Returning to pharmacy	60 (15)
Donation to hospitals	11 (2.8)
Giving to friends/family	7 (1.8)
None recommended (I don’t know)	23 (5.8)
Other	6 (1.5)
**Total**	**400 (100)**
**Liquid drugs**	
Trash	131 (32.8)
Toilet	43 (10.8)
Sink	132 (33)
Incineration	3 (0.8)
Returning to pharmacy	50 (12.5)
Donation to hospitals	7 (1.8)
Giving to friends/family	6 (1.5)
None recommended (I don’t know)	23 (5.8)
Other	5 (1.3)
**Total**	**400 (100)**
**Class-B drugs**	
Trash	82 (20.5)
Toilet	17 (4.3)
Sink	2 (0.5)
Incineration	23 (5.8)
Returning to pharmacy	191 (47.75)
Donation to hospitals	12 (3)
Giving to friends/family	2 (0.5)
None recommended (I don’t know)	48 (12)
Other	23 (5.8)
**Total**	**400 (100)**
**Class-C drugs**	
Trash	86 (21.5)
Toilet	19 (4.8)
Sink	4 (1)
Incineration	19 (4.8)
Returning to pharmacy	195 (48.8)
Donation to hospitals	5 (1.3)
Giving to friends/family	2 (0.5)
None recommended (I don’t know)	46 (11.5)
Other	24 (6)
**Total**	**400 (100)**
**Antibiotics**	
Trash	266 (66.5)
Toilet	22 (5.5)
Sink	2 (0.5)
Incineration	8 (2)
Returning to pharmacy	56 (14)
Donation to hospitals	4 (1)
Giving to friends/family	5 (1.3)
None recommended (I don’t know)	28 (7)
Other	9 (2.3)
**Total**	**400 (100)**
**Hormonal drugs**	
Trash	176 (44)
Toilet	19 (4.8)
Sink	2 (0.5)
Incineration	6 (1.5)
Returning to pharmacy	148 (37)
Donation to hospitals	2 (0.5)
Giving to friends/family	4 (1)
None recommended (I don’t know)	37 (9.3)
Other	6 (1.5)
**Total**	**400 (100)**
**Inhaler drugs**	
Trash	171 (42.8)
Toilet	17 (4.3)
Sink	5 (1.3)
Incineration	11 (2.8)
Returning to pharmacy	114 (28.5)
Donation to hospitals	3 (0.8)
Giving to friends/family	4 (1)
None recommended (I don’t know)	66 (16.5)
Other	9 (2.3)
**Total**	**400 (100)**

### Methods of disposal recommended by pharmacists to patients or the general public

Most pharmacists in our study (252, 63%) advised their patients to dispose of unwanted solid drugs in the trash, 78 (19.5%) advised patients to return these drugs to the pharmacy, and 19 (4.8%) reported not knowing what to tell patients when asked. (Table 2).

For semisolid drugs, most pharmacists in our study (196, 49%) advised their patients to dispose of them in the trash. Most pharmacists in our study advised their patients to dispose of liquid drugs through the sink (132, 33%) or trash (131, 32.8%). Regarding class-B drugs, most participants (191, 47.7%) advised their patients to return to the pharmacy. Regarding class C drugs, most participants (195, 48.8%) advised their patients to return them to the pharmacy. Regarding antibiotic disposal, the vast majority of study participants (266, 66.5%) recommended the disposal of these drugs in the trash. Regarding hormonal drugs, the majority of participants recommended throwing them away in the trash (176, 44%) or returning them to the pharmacy (148, 37%).

Most participants recommended that inhaler drugs be disposed of in the trash (171, 42.8%) or returned to the pharmacy (114, 28.5%). However, 66 (16.5%) did not know what to tell patients when asked, and 17 (4.3%) recommended that they be disposed of in the bathroom (Table 2).

### Pharmacists’ awareness regarding the environmental hazards of improper drug disposal

Most pharmacists agreed (245, 61.3%) or strongly agreed (103, 25.8%) that drug disposal through the trash, sink, or toilet may cause environmental harm. Additionally, most agreed (217, 54.3%) or strongly agreed (84, 21%) that unsafe drug disposal practices can contribute to increasing antibiotic resistance. Similarly, most participants agreed (218, 54.5%) or strongly agreed (74, 18.5%) that unsafe drug disposal may contribute to hormone-dependent cancers (Table 3).

**Table 3:** Pharmacists’ awareness regarding the environmental hazards of improper drug disposal

Question		Strongly agree		Agree		Neither agree or disagree		Disagree		Strongly disagree		Total	
		n	%	n	%	n	%	n	%	n	%	n	%
**1**	There are environmental harms that can occur due to drug disposal via trash or sink or toilet	103	25.8	245	61.3	38	9.5	13	3.3	1	0.3	**400**	**100**
**2**	Unsafe drug disposal practices may be a contributing factor to increasing antibiotic resistance	84	21	217	54.3	62	15.5	31	7.8	6	1.5	**400**	**100**
**3**	Unsafe drug disposal practices may be a contributing cause of hormone-dependent cancers	74	18.5	218	54.5	83	20.8	21	5.3	4	1	**400**	**100**
**4**	Community pharmacists ensure that expired drugs are properly separated and sorted in the correct way	102	25.5	229	57.3	54	13.5	12	3	3	0.8	**400**	**100**
**5**	It is my responsibility as a pharmacist to protect the environment, even if others are unconcerned or are irresponsible	142	35.5	233	58.3	18	4.5	6	1.5	1	0.3	**400**	**100**
**6**	It is my responsibility to ensure the safety of other living creatures on earth	133	33.3	184	46	75	18.8	5	1.3	3	0.8	**400**	**100**
**7**	Perceived sources of unwanted medications	**Returned from patients**		**Returned from hospitals**		**Leftovers from labs**		**Other**		**Total**			
		**n**	**%**	**n**	**%**	**n**	**%**	**n**	**%**	**n**		**%**	
		321	80.3	17	4.3	19	4.8	43	10.8	**400**		**100**	

Most participants believed that community pharmacists should ensure that expired drugs are properly sorted and separated, with 229 (57.3%) agreeing and 102 (25.5%) strongly agreeing with the statement. Most also agreed (233, 58.3%) or strongly agreed (142, 35.5%) that pharmacists are responsible for protecting the environment, even if others remain unconcerned or impartial. A smaller number of pharmacists agreed (184, 46%) or strongly agreed (133, 33.3%) that it was their responsibility to ensure other living creatures’ safety.

Regarding the sources of these unwanted medications, most pharmacists (321, 80.3%) saw that they were returned from patients. Other perceived sources with fewer contributions included those returned from hospitals (17, 4.3%), leftovers from laboratories (19, 4.8%), and other sources (43, 10.8%).

### Pharmacists’ views on funding for expired-drug disposal systems

The vast majority of participants in our study (389, 97.3%) believed that Palestine needs an easily accessible national drug disposal system. Most (310, 77.5%) thought district health boards should fund this. Meanwhile, 77 (19.3%) believed that pharmaceutical companies should fund it (Table 4).

**Table 4:** Pharmacists’ views on funding for expired-drug disposal systems

Question	n (%)
**Do you think Palestine needs a national drug disposal system that is easily accessible from all pharmacies in the country?**	
No	11 (2.8)
Yes	389 (97.3)
**Who should fund drug-disposal systems?**	
Patients	5 (1.3)
The district health board	310 (77.5)
Pharmaceutical companies	77 (19.3)
Community pharmacies	8 (2)
**Total**	**400 (100)**

### Pharmacists’ views on recommendations to improve safe drug-disposal practices

Providing proper training to healthcare workers on managing expired medications was the most favored recommendation among participants in our study to improve drug disposal practices, with 206 pharmacists (51.5%) agreeing and 176 pharmacists (44%) strongly agreeing with the recommendation. Organizing events and scientific forums around proper drug disposal was the second most preferable recommendation. The distribution of brochures on safe drug disposal was the third most popular recommendation, with 244 (61%) agreeing and 133 (33.3%) strongly agreeing with this idea. The least common proposal was the passage of strict laws on the safe disposal of all forms of drugs (Table 5).

**Table 5:** Pharmacists’ views on recommendations to improve safe drug-disposal practices

	Recommendation	Strongly agree		Agree		Neither agree or disagree		Disagree		Strongly disagree		Total	
		**n**	**%**	**n**	**%**	**n**	**%**	**n**	**%**	**n**	**%**	**n**	**%**
**1**	Putting containers for unwanted drugs in the pharmacy and assigning a day of the year to collect and dispose of these medications	141	35.3	230	57.5	21	5.3	8	2	0	0	**400**	**100**
**2**	Pass strict laws regarding the safe disposal of all forms of drugs	129	32.3	181	45.3	84	21	6	1.5	0	0	**400**	**100**
**3**	Organizing events and scientific forums, including conferences and workshops, around the proper disposal of unwanted drugs	141	35.5	240	60	18	4.5	1	0.3	0	0	**400**	**100**
**4**	Distribution of brochures on safe drug disposal to encourage pharmacists to read and stay up to date on the topic continually	133	33.3	244	61	19	4.8	3	0.8	1	0.3	**400**	**100**
**5**	Providing proper training to healthcare workers regarding the management of expired medications	176	44	206	51.5	16	4	2	0.5	0	0	**400**	**100**

### Pharmacists’ views on common challenges facing drug disposal processes

Participants in our study viewed the greatest challenge facing drug disposal processes as poor education and awareness about the management of expired or unwanted drugs, and 339 (84.8%) agreed with this statement. Other notable challenges perceived by study participants included poor law enforcement (265, 66.3%), difficulty returning drugs to disposal sites due to lack of formal policy on the matter (238, 59.5%), and lack of documentaries on the management of expired or unwanted drugs (163, 40.8%).

Meanwhile, 88 pharmacists (22%) saw laws regulating the transport of controlled drugs as an obstacle to the proper drop-off and disposal of these drugs, and 20 pharmacists (5%) saw that there were other challenges to the process (Table 6).

**Table 6:** Pharmacists’ views on common challenges facing drug disposal processes

Perceived challenges	Number of pharmacists who agreed (%)
Poor education and awareness about the management of expired or unwanted drugs	339 (84.8)
Lack of documentaries on the management of expired or unwanted drugs	163 (40.8)
Poor enforcement of the law	265 (66.3)
Laws regulating the transport of controlled drugs are an obstacle to the return of these drugs and their proper disposal.	88 (22)
The difficulty of returning drugs to disposal sites due to the lack of facilities and local capabilities	139 (34.8%)
The difficulty of returning drugs to disposal sites due to limitations on drop-off times	121 (30.3)
The difficulty of accessing drug drop-off sites is an obstacle to proper drug disposal.	121 (30.3)
The difficulty of returning drugs to disposal sites due to the lack of formal policy on the matter	238 (59.5)
Other	20 (5)

### Correlations between awareness score and pharmacists’ characteristics

The median [Q1-Q3] awareness score was 24.00 [23.00–26.00]. The univariate analysis results showed that pharmacists with higher education degrees were more aware of the disposal of unwanted pharmaceutical products. Furthermore, participants who previously participated in courses on proper drug disposal methods had higher scores than others (Table 7).

**Table 7:** Correlations between pharmacists’ characteristics and awareness

Variable	Median [Q1-Q3]	P value
**Sex**		0.289*
Male	24.00 [23.00–25.00]	
Female	24.00 [23.00–27.00]	
**Pharmacy location**		0.745**
City	24.00 [23.00–26.00]	
Village	24.00 [23.00–26.00]	
Refugee camp	24.00 [23.00–24.00]	
**Job title**		0.392*
Pharmacist	24.00 [23.00–26.00]	
Responsible pharmacist	24.50 [21.00–26.00]	
**Pharmacists’ residence**		0.603**
City	24.00 [23.00–26.00]	
Village	24.00 [23.00–26.00]	
Refugee camp	24.00 [23.75-26.00]	
**City**		0.060**
Bethlehem	23.00 [21.00-23.50]	
Nablus	24.00 [21.00–26.00]	
Ramallah	27.00 [25.00–28.00]	
Jerusalem	-	
Jenin	24.00 [23.00–25.00]	
Tulkarem	24.50 [22.00–25.00]	
Salfit	24.00 [21.50–25.00]	
Qalqilya	24.00 [22.00–26.00]	
Tubas	26.00 [24.00-26.50]	
Hebron	24.00 [23.00–27.00]	
Other	27.00 [22.00–29.00]	
**Marital status**		0.065*
Married	24.00 [23.00–27.00]	
Single	24.00 [23.00–25.00]	
**Academic qualifications**		0.022**
Bachelor of pharmacy	24.00 [23.00–26.00]	
Doctor of pharmacy	23.00 [22.00–25.00]	
Higher education (post-grad)	25.00 [22.00–27.00]	
**Country of graduation**		0.907*
Palestine	24.00 [23.00–26.00]	
Abroad	25.00 [21.75-26.00]	
**Monthly income**		0.117**
Less than 400 US dollar	25.00 [23.00–27.00]	
400–800 US dollar	24.00 [23.00–26.00]	
Over 800 US dollar	25.00 [22.00–26.00]	
**Previous participation in courses on proper drug disposal methods**		0.026*
Yes	25.00 [24.00–27.00]	
No	24.00 [23.00–26.00]	

* Mann‒Whitney U test

** Kruskal‒Wallis test

## Discussion

In this study, out of 400 community pharmacists, 293 (73.3%) reported that they disposed of unwanted drugs by returning them to manufacturing companies and warehouses. Similar results were found in a study conducted in Saudi Arabia, where between 73.3% and 75.3% reported that the disposal of unused medication was mainly through pharmaceutical suppliers [32]. Another similar study in New Zealand showed that the main disposal route was managed by specialized third-party contractors (80%) [4]. However, a survey study conducted in Kuwait among community pharmacists found that most (73%) respondents disposed of unused medications in the trash and the sink [30]. The disposal of drugs in the trash was used as a second method of drug disposal (48.3%) in our study. This practice of disposing of unused medications in the trash or through the sink caused 97.3% of community pharmacists to support establishing a drug disposal system in Palestine, 77.5% of whom chose to have the district health board responsible for financing this system. In the New Zealand study, 90% of respondents agreed that health authorities should establish a destruction system for unused medications [4].

The current study showed that 61.3% of pharmacists agreed and 26% strongly agreed that unsafe disposal of drugs negatively affects the environment. These results were similar to those found in Saudi Arabia, as 79% of community pharmacists agreed that inappropriate disposal of unused medication might negatively impact the environment [32]. Furthermore, most of them agreed or strongly agreed and considered themselves responsible for protecting the environment (93.8%) by taking the role of collecting unused medications; related studies in Kuwait and Saudi Arabia revealed similar results [30, 32].

Community pharmacists in Palestine showed a good level of awareness regarding inappropriate practices of medication disposal, and the median [Q1-Q3] awareness score was 24.00 [23.00–26.00], which is similarly reported by a study in Saudi Arabia in which community pharmacies considered themselves responsible for protecting the environment by proper disposal practices [32]. The awareness level was positively correlated with the education level of pharmacists, which is expected, as the higher the level of education, the more courses covered safe disposal the pharmacist received. This is also shown in a study conducted in Iraq, where the majority of pharmacists (85.3%) agreed that advanced education courses and programs will help raise awareness about proper medication disposal [29].

Furthermore, participants who previously participated in courses on proper drug disposal methods had higher awareness scores than others. In these courses, pharmacists received education on the proper disposal practices, which eventually positively affected their level of awareness. These findings are similarly supported by studies conducted in New Zealand, Kuwait and Saudi Arabia that strongly encouraged conducting education courses and training programs for pharmacists and other healthcare professionals as the best way to increase their level of awareness [27, 30, 32].

All the results and reasons mentioned above support the need to establish policy guidelines for the safe disposal of medications within community pharmacies in Palestine. However, to achieve efficient and effective results using safe disposal, barriers and challenges facing community pharmacists in Palestine reported in this study must be solved, such as the lack of education and awareness on the issue of removing unused drugs and weak law enforcement. Most participants agreed that good training for health care professionals through organizing workshops to develop knowledge and awareness of this subject would be an important step in improving practices.

In this article, we found that some pharmacists recommended that patients donate medications to hospitals or give them to family/friends. Although some countries may have legal restrictions on such practices, there are no restrictions or guidance about this practice in Palestine. The questionnaire included these choices because some pharmacists may recommend these practices under certain circumstances. For example, it could be cost-effective and environmentally friendly to donate unwanted medications instead of disposing of them [33].

The results also showed that approximately 20% of pharmacists return unwanted medications to the Ministry of Health. The reason beyond that is to ensure the safe and proper disposal of certain drugs, such as controlled substances.

This study was carried out on the West Bank in Palestine, and most of the sample was from the North Regions, which is considered a limitation of our study. Furthermore, the current study focuses on community pharmacists, although the problem of unwanted medication disposal affects other healthcare professionals, households, and healthcare institutions. In addition, our study was generalized, assessing awareness and practices of unwanted pharmaceutical products and does not specify dangerous or controlled drugs or quantities. However, environmental or human harm could occur from any type or quantity of drug if disposed of improperly.

## Conclusions

Most pharmacists in our study returned drugs to manufacturing companies and stores, approximately half of them disposed of drugs in the trash, and few followed the correct incineration and return of drugs to the Ministry of Health. Current data emphasize the improper disposal of medicine in Palestine and the need for improved education among healthcare workers. Further research is needed to answer the question about the quantities of pharmaceuticals discarded by different sectors and their relation to the total amount of drugs discharged in Palestine. Specifically, we recommend using the simulated client technique that gives a better picture of practice assessment as well as decreases bias in results.

## Data Availability

Data collected and analysed for this study are available from the corresponding author upon reasonable request.

## List of abbreviations

FDA: Food and Drug Administration
IQR: Interquartile range
IRB: Institutional Review Board
NIS: New Israeli Shekel
SPSS: The Statistical Package for Social Sciences
